# Supplementary material for: Association between oral microbiome and seven types of cancers in East Asian population: a two-sample Mendelian randomization analysis
Source: Front Mol Biosci. 2023 Nov 21;10:1327893. doi: 10.3389/fmolb.2023.1327893 (PMC10702768; doi:10.3389/fmolb.2023.1327893)
Supplement: Supplementary file 1 [file DataSheet1.ZIP › Supplementary Materials/MR plots for tongue/tongue═╝/Pancreatic cancer/╥╚╧┘░⌐_╔╕╤í╜ß╣√/pheno.225_to_pancreatic cancer_forest.pdf]

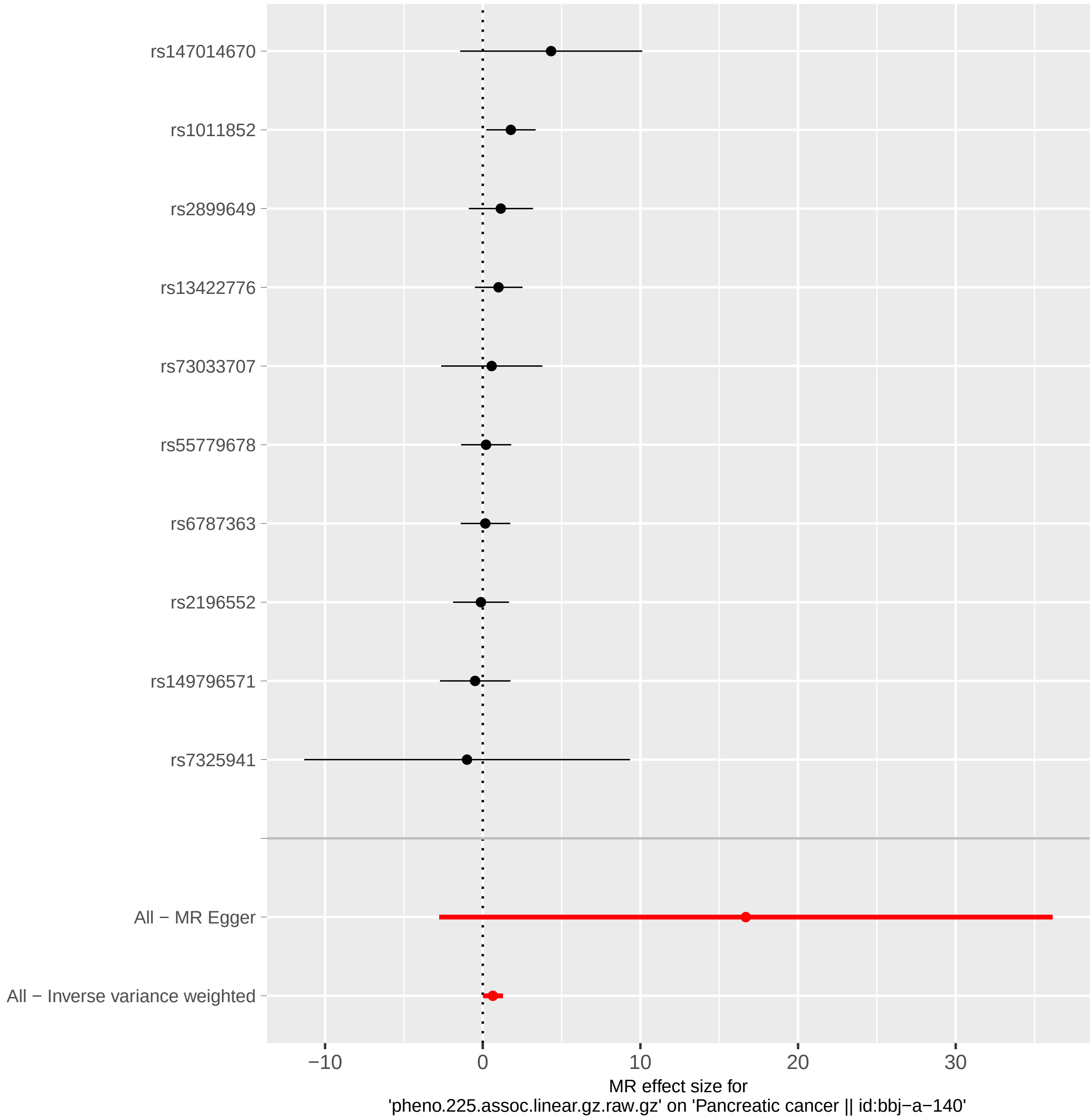

rs147014670
rs1011852
rs2899649
rs13422776
rs73033707
rs55779678
rs6787363
rs2196552
rs149796571
rs7325941
All − MR Egger
All − Inverse variance weighted
−10
0
10
20
30
MR effect size for
'pheno.225.assoc.linear.gz.raw.gz' on 'Pancreatic cancer || id:bbj−a−140'
